# High dissimilarity within a multiyear annual record of pollen assemblages from a North American tallgrass prairie

**DOI:** 10.1002/ece3.2259

**Published:** 2016-06-29

**Authors:** Julie L. Commerford, Kendra K. McLauchlan, Thomas A. Minckley

**Affiliations:** ^1^ Department of GeographyKansas State UniversityManhattanKansas66506; ^2^ Department of GeographyUniversity of WyomingLaramieWyoming

**Keywords:** Fire, grassland, Great Plains, herbivory, pollen, Tauber traps

## Abstract

Grassland vegetation varies in composition across North America and has been historically influenced by multiple biotic and abiotic drivers, including fire, herbivory, and topography. Yet, the amount of temporal and spatial variability exhibited among grassland pollen assemblages, and the influence of these biotic and abiotic drivers on pollen assemblage composition and diversity has been relatively understudied. Here, we examine 4 years of modern pollen assemblages collected from a series of 28 traps at the Konza Prairie Long‐Term Ecological Research Area in the Flint Hills of Kansas, with the aim of evaluating the influence of these drivers, as well as quantifying the amount of spatial and temporal variability in the pollen signatures of the tallgrass prairie biome. We include all terrestrial pollen taxa in our analyses while calculating four summative metrics of pollen diversity and composition – beta‐diversity, Shannon index, nonarboreal pollen percentage, and *Ambrosia:Artemisia* – and find different roles of fire, herbivory, and topography variables in relation to these pollen metrics. In addition, we find significant annual differences in the means of three of these metrics, particularly the year 2013 which experienced high precipitation relative to the other 3 years of data. To quantify spatial and temporal dissimilarity among the samples over the 4‐year study, we calculate pairwise squared‐chord distances (SCD). The SCD values indicate higher compositional dissimilarity across the traps (0.38 mean) among all years than within a single trap from year to year (0.31 mean), suggesting that grassland vegetation can have different pollen signatures across finely sampled space and time, and emphasizing the need for additional long‐term annual monitoring of grassland pollen.

## Introduction

Grasslands occupy 24% of Earth's land area and contribute to global food production through row‐crop agriculture and grazing or pasture lands. Because grasslands experience high spatial and temporal variability in climate, they are predicted to be especially vulnerable to future climate change (IPCC 2014). Grasslands in North America are particularly at risk because they are currently spatially restricted and regularly experience severe droughts such as the Dust Bowl of the 1930s (Cook et al. 2010). Other similar long‐term drought events have occurred in this region throughout the Holocene (the past 11,500 years), changing the species composition and extent of this biome (Clark et al. 2001; Umbanhowar et al. 2006; Grimm et al. 2011). However, the precise timing of these changes and the factors influencing those changes at various spatial scales are still unknown. Therefore, both the composition and biodiversity of grasslands over time and the role that biotic and abiotic factors have played in structuring them remain unanswered questions. A long‐term perspective is essential to capture the slow processes thought to be important in North American grasslands, such as megadroughts, grazing from large herbivores, and changes in fire regimes.

Pollen from lacustrine sediment cores records how vegetation responded to those processes on relevant timescales of several decades to millennia (Clark et al. 2001; Brown et al. 2005). While pollen from sediment cores can provide a unique source of information about past vegetation on a landscape, it has been difficult to obtain quantitative estimates of vegetation cover. The main reason for this problem is that pollen is not produced in proportion to plant abundance on the landscape (Sugita 1994). One way to overcome this limitation is by calibrating modern pollen assemblages with site features such as vegetation, climate, fire, herbivory, or topography. For example, much work has been made to calibrate pollen with vegetation by calculating pollen productivity of select plant taxa in Europe (Soepboer et al. 2007; Brostrom et al. 2008; Mazier et al. 2008) and in the United States (Sugita et al. 2006; Commerford et al. 2013). In addition, calibrations of pollen with climate have been conducted in a variety of ecosystems, including grasslands (Gajewski et al. 2002; Minckley et al. 2008; Tonello and Prieto 2008).

**Figure 1:**
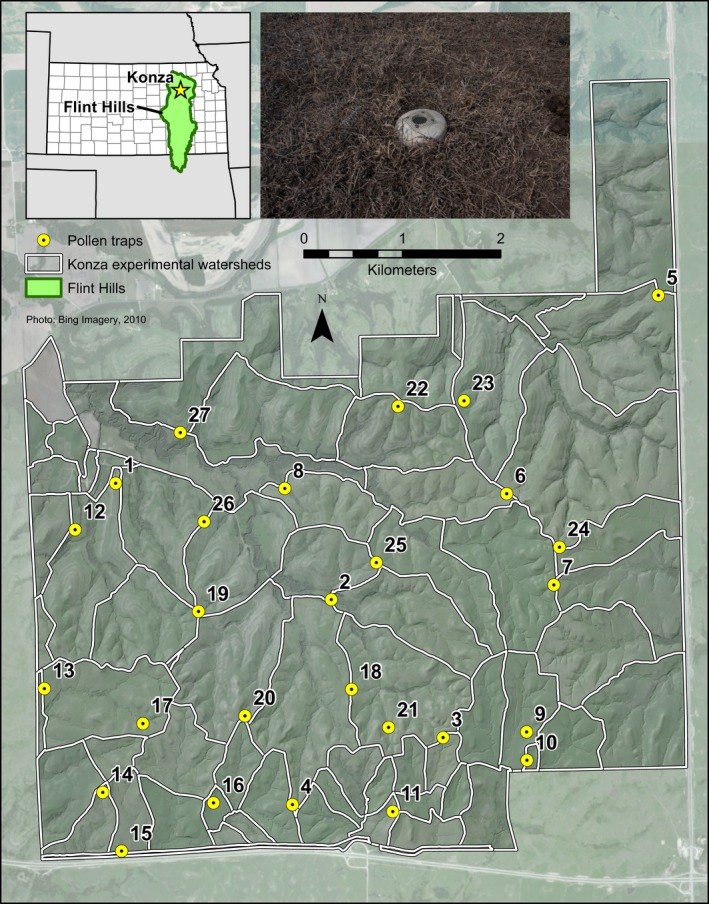
Trap locations within the Konza Prairie Long‐Term Ecological Research Area. Konza is located within the Flint Hills ecoregion (inset map). A typical trap is pictured in the photograph.

Modern pollen samples are acquired in several ways, including surface sediments from lakes or ponds, moss polsters, or Tauber traps (Tauber 1974). Tauber traps have been successfully deployed over a variety of forested landscapes in Poland, Norway, Finland, Latvia, Czech Republic, Switzerland, Greece, and Bulgaria in multiyear studies of pollen production through the European Pollen Monitoring Programme (Giesecke et al. 2010). The goal of these studies has been to calibrate pollen deposition with vegetation cover and examine annual and seasonal variability (Hicks 1985, 2001; Räsänen et al. 2004; Giesecke and Fontana 2008; Bjune 2014). Tauber traps are typically made of thick plastic, are cylindrical in shape, and are approximately 25 cm tall by 15 cm in diameter (Fig. 1). An opening of 5 cm in diameter at the top of the trap allows pollen to enter and remain trapped for the duration of the sampling period.

Two recent examples from Europe demonstrate the significant value of these multiyear pollen records. In a birch‐pine forest in Norway, nine Tauber traps were used to collect pollen annually from 2004 to 2012 (Bjune 2014). After 8 years of sampling, there was higher interannual variability in pollen productivity than after only 2 years of sampling. These results suggested that multiyear, annual records of pollen deposition are necessary to understand the degree of variability in pollen representing a landscape over time. In a similar study in a birch forest in Finland, 6 years of pollen at four different Tauber traps were collected and compared with corresponding years of meteorological data (Autio and Hicks 2004). Here, the deposition of *Pinus* (pine) pollen was positively correlated with July temperature. These findings demonstrated that climate conditions, particularly temperature, could be used to predict pollen production for the following year. These studies of annual pollen production reveal the need for caution in overinterpreting environmental conditions at the onset of changes in paleoenvironmental records as increases in individual pollen taxa could be responses to rapid shifts in climate (Minckley et al. 2012a).

While multiyear studies have been important for improving vegetation and climatic interpretations from pollen, to our knowledge there have been few similar studies in North America. One North American study examined annual modern grassland pollen records, but focused on spatial differences among grassland types and only collected pollen for one or 2 years (Hoyt 2000). Most importantly, little work has been made to calibrate factors aside from climate or vegetation, such as fire, herbivory, or topography. These factors are of major importance in grasslands (Knapp et al. 1999; Craine and McLauchlan 2004; Grimm et al. 2011). Consequently, there is a clear need to understand the quantitative relationships among fire, herbivory, and topography and present‐day grassland composition. A detailed examination of how those factors influence pollen deposition on an annual basis is required to understand these relationships in the distant past.

Here, we present a 4‐year annual record of modern pollen assemblages from a tallgrass prairie in Kansas, with the aim of quantifying temporal and spatial variation in the context of biotic and abiotic drivers. We evaluate annual grassland pollen assemblages with several different fire, herbivory, and topography variables to achieve three main objectives: (i) assess the influence of these variables on the pollen assemblages; (ii) quantify the degree of interannual variability among the pollen assemblages; and (iii) determine whether the pollen assemblages exhibit higher spatial variation (between sample locations) or temporal variation (within sample locations). To do this, we calculate four summative metrics from each pollen assemblage to represent their composition and/or diversity, and then quantify the degree of explanation of each of these metrics by the environmental variables. We then statistically compare the differences in these metrics across the 4 years of sampling. Finally, we evaluate the degree of dissimilarity between pollen assemblages across the study area and through time by calculating squared‐chord distance between each pollen assemblage and all other assemblages. By doing so, we provide greater insight into the factors that drive grassland pollen assemblage composition and diversity, as well as highlight the amount of variation that exists among grassland pollen assemblages across space and through time.

## Materials and Methods

### Study area

Konza Prairie Long‐Term Ecological Research Area (hereafter “Konza”) is a 3487‐hectare preserve of native tallgrass prairie in the Flint Hills of northeastern Kansas (Fig. 1). The Flint Hills contains the largest contiguous unplowed tract of prairie remaining in North America (Knapp et al. 1999), and the parent material consists of alternating layers of limestone and mudstone. Konza is divided into watershed‐level experimental units that were delineated in 1981, and each unit has a specified grazing and burning treatment. Each watershed is burned every 1, 2, 3, 4, or 20 years. A herd of approximately 300 native bison have resided on Konza since its introduction in 1987, and are restricted to the area outlined in black on Figure 1.

The preserve is floristically diverse, with 597 different plant species: 86 species of grasses, 409 species of forbs, and 59 woody species (Towne 2002). Konza was chosen for this study for a number of reasons: (i) It has never been plowed and so is the best present‐day proxy for grasslands prior to the onset of modern agriculture; (ii) Its floristic diversity provides an ideal location for capturing differences in pollen representation among grassland plants; (iii) The different burning and grazing treatments facilitate direct comparison between pollen assemblages in watersheds that are burned at different frequencies, in addition to grazed versus ungrazed areas; and (iv) Its purpose as a long‐term ecological research area enables a multiyear study where pollen traps can remain in‐place for several years with minimal disruption.

### Data collection

Twenty‐eight modified Tauber traps (Tauber 1974) were placed on Konza in October 2008. Traps were placed stratified‐randomly to cover both grazed and ungrazed areas, different burning treatments, and upland and lowland areas. Traps were placed at least 500 m apart. The trap contents were collected in October 2009, 2010, 2011, 2012, 2013, and 2014 (at the end of the growing season). Each trap was rinsed in the field with deionized water and poured into an empty jug. In the laboratory, the contents were filtered through cellulose paper and soaked with glacial acetic acid to prevent the growth of mold or fungi. Each filter paper was processed and prepared for pollen analysis using standard techniques (Faegri and Iversen 1989) with a modification made for dissolving the filter paper based on the European Pollen Monitoring Programme protocol. Each pollen sample was mounted in silicone oil, examined under a light microscope, and counted to a minimum sum of 300 terrestrial grains. All pollen grains were identified to the finest possible taxonomic resolution, generally following McAndrews et al. (1973). This article focuses specifically on the pollen data collected in 2011, 2012, 2013, and 2014. The pollen data from 2009 and 2010 are published in Gill et al. (2013).

To evaluate the effects of fire, grazing, and topography on the pollen assemblages, we measured several different environmental variables at each sample location. Most environmental variables for each sample location were obtained from Leys et al. (2015), which examined macroscopic charcoal from this same set of traps for the same years (2011–2014). The fire metrics we included were mean fire return interval, fire return interval, fire frequency, time since last fire, regional area burned within the Flint Hills ecoregion, local area burned within Konza, and area burned within 500 m of the trap. Topography metrics we included from Leys et al. (2015) were elevation, presence of bare soil around the trap, presence of bison manure (as a qualitative proxy for presence/absence of bison), and presence of limestone rocks (within 500 m of the trap). To supplement the above variables, we used bison density data calculated as amount of time spent by bison within 500 m of each trap via GPS collars and previously reported in Gill et al. (2013). We also calculated total precipitation and mean average, maximum, and minimum temperature for the months May through August for each year, based on data from the Konza Prairie weather station (KPBS 2015), to examine any covariance of these factors with the 4 years of pollen data. All three temperature variables were found to exhibit no significant annual differences for the 4 years covered in this study and so were not pursued further. Precipitation was included because of significant annual differences.

### Data analysis

Raw pollen counts were converted to percentages using a pollen sum that included all terrestrial taxa and analyzed in Tilia 2.0 (Grimm 1993). High variability in volume of water used to wash the traps and filter their contents precluded calculations of absolute pollen influx rate, although the protocol could be amended in future years to allow that calculation. One trap was excluded from the analyses because it contained no pollen in 2011 and 2012. To initially examine the degree to which the environmental variables influenced the multivariate pollen samples, we conducted a canonical correspondence analysis (CCA) (Ter Braak 1986) using Canoco 5 Software (Ter Braak and Smilauer 2012). The CCA suggested some influence of certain environmental variables on the multivariate pollen assemblages, but overall low explanation (highest eigenvalue = 0.06). Thus, we determined that a collective value of diversity or composition for each sample based on all of the pollen taxa would be more useful for directly assessing the influence of the environmental variables. We chose to summarize each pollen sample (each trap from each year) using four different metrics included in a regression analysis and compared across all 4 years. The four different metrics calculated were as follows: (i) Shannon index, (ii) beta‐diversity, (iii) nonarboreal pollen percentage, and (iv) ratio of *Ambrosia:Artemisia* pollen. Shannon index was calculated using the standard equation for Shannon diversity (Equation 1), where *p* is the proportion of the sample belonging to pollen taxa *i*.

$$H^{\prime }=-\sum_{i=1}^{R}p_{i}\ln p_{i}$$

### Equation 1**:** Shannon index

Shannon index has been found to be a robust measure of vegetation diversity when used on pollen assemblages (Matthias et al. 2015). Beta‐diversity was calculated via a detrended canonical correspondence analysis (DCCA), constrained by year. This metric computes the ratio between the sample diversity at a given trap for 1 year compared to the sample diversity at that same trap for the other 3 years. Nonarboreal pollen percentage was calculated by totaling the abundance proportions of all grass and forb pollen in each sample. *Ambrosia:Artemisia* was calculated as the ratio of *Ambrosia:Artemisia* pollen percentages in each sample and has been suggested to be an indicator of grassland type (tallgrass vs. shortgrass) (Morris 2013).

To assess which environmental variables influenced each of the four summative pollen metrics, individual multiple regressions were performed. All summative metrics and environmental variables were rescaled between 0 and 1 using the “scales” package in R (Wickham 2016). Each regression used one of the four summative metrics (Shannon index, beta‐diversity, nonarboreal percentage, and *Ambrosia:Artemisia*) as the response variable, and all of the previously mentioned environmental variables as the explanatory fixed‐effect variables. Random effects for trap and year were also included in each regression model and tested for significance. For each response variable, this was performed by creating a full model of all fixed effects (environmental variables) and random effects (for trap and year). Then, the random effects of trap and year were each removed individually from the full model, and the resulting model was statistically compared to the full model. The regression models were built using lme4 package (Bates et al. 2015) in R. Comparisons between the full models and the models with random effects removed were conducted using the stats package in R (R Core Team 2015). Please see Appendix S1 for specific code.

We also compared differences in the summative metrics across all 4 years of data. To do this, a Kruskal–Wallis ANOVA test was conducted on each of the four metrics across the 4 years, to determine whether the means for each year were significantly different overall (at *P *< 0.05). A Kruskal–Wallis *post hoc* pairwise comparison tested for significant differences between the mean of each year and each of the other 3 years (at *P *< 0.05). Finally, an analysis of covariance (ANCOVA) was conducted to test the covariance of total growing season precipitation and year for each of the four summative metrics. The temperature variables mentioned earlier (minimum, average, and maximum temperature) were excluded from the analysis because they were not significantly different across the 4 years of data. All of these analyses were conducted in R statistical software (R Core Team 2015), using the stats package for the regression and ANCOVA (R Core Team 2015), and the PGIRMESS package (Giraudoux 2014) for the ANOVAs and post hoc comparisons. Please see Appendix S1 for specific code.

To evaluate the dissimilarity between each pollen sample, we calculated pairwise squared‐chord distances. The squared‐chord distance (SCD) metric (Overpeck et al. 1985) incorporates all pollen taxa in each sample and essentially computes the component distance between sample pairs, such that pairs with higher SCD values are more dissimilar, while pairs with lower SCD values are more similar. SCD has long been used in pollen studies to detect a matching modern pollen sample for a fossil pollen sample (Williams et al. 2009; Minckley et al. 2012b; McLauchlan et al. 2013). It has also been used to compare dissimilarity among pollen samples across a modern study area (Hoyt 2000; Gavin et al. 2005; Minckley et al. 2008), such as in this study. We calculated SCD in R Statistical Software (R Core Team 2015), using the Analogue package (Simpson 2015). Please see Appendix S1 for specific code.

## Results

### Pollen assemblages among traps and years

**Figure 2:**
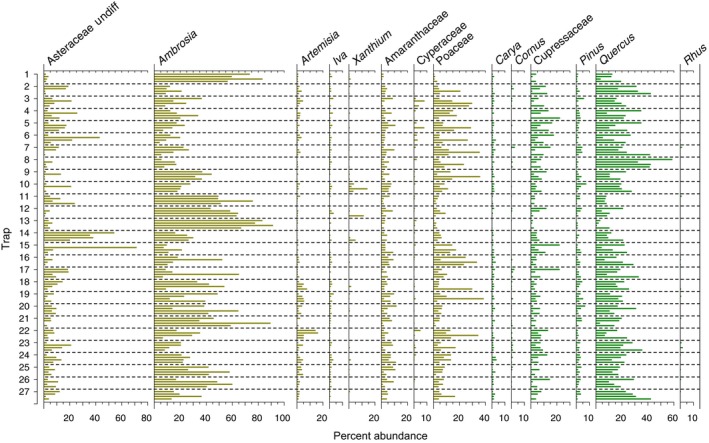
Pollen abundance (%) for the 6 most abundant taxa and other key taxa. Nonarboreal taxa are shown in brown, and arboreal taxa are shown in green. For each trap, each bar signifies one collection year, beginning with 2011 at the top and 2014 at the bottom.

Percent abundance of pollen taxa varies significantly among the 27 Tauber traps at the Konza study area from 2011, 2012, 2013, and 2014 (Fig. 2). The number of different pollen taxa from each trap/year ranges from 11 to 32, averaging 22 taxa. The six most abundant pollen taxa found in the traps on average are (in order from the highest): *Ambrosia* (31.1%)*, Quercus* (18.9%)*,* Poaceae (9.6%), Undifferentiated Asteraceae (8.9%), Cupressaceae (5.7%)*,* and Amaranthaceae (4.1%). Four of these taxa are nonarboreal (*Ambrosia,* Poaceae, undifferentiated Asteraceae, and Amaranthaceae), and two of these taxa are arboreal (*Quercus* and Cupressaceae).

The identity of the most abundant pollen taxa varies locally from trap to trap, although certain traps exhibit more variation across the 4 years of data than other traps. At Trap 2 and Trap 8, *Quercus* is consistently the most abundant taxa for all 4 years of data. Conversely, at Trap 1, 11, 12, 13, 21, and 25, *Ambrosia* is consistently the most abundant taxa for all 4 years. In addition, some of the less abundant (but still common) taxa also exhibit trends. *Xanthium* pollen is higher at Trap 10 than at any of the other traps for 2011, 2012, and 2013. *Artemisia* is highest in 2011, 2012, and 2013 at Trap 22 compared to all the other traps but is higher at Trap 17 in 2014. Thus, trap locations contribute to the types of pollen captured.

### The role of environmental variables on pollen metrics

Overall, the four summative pollen metrics – beta‐diversity, Shannon index, nonarboreal percentage (NAP%), and *Ambrosia:Artemisia* – are each partially influenced by different environmental variables (Table 1). Some environmental variables (fixed effects) significantly influence more than one of the summative metrics. For example, the area burned within the Flint Hills contributes to both beta‐diversity and NAP%. Shannon index is influenced by bison density. Beta‐diversity is influenced by the area burned within the Flint Hills and the area burned within Konza. NAP% is influenced by area burned within the Flint Hills and area burned within Konza. *Ambrosia:Artemisia* is influenced by the area burned within 500 m of the trap. The random effects of trap location and year also vary in significance for the four summative metrics. Trap location significantly influences Shannon index, NAP%, and *Ambrosia:Artemisia*. Year significantly influences Shannon index only. Beta‐diversity is not significantly influenced by trap location or year.

**Table 1:** Multiple regression results of the four summative metrics listed at top (beta‐diversity, Shannon index, nonarboreal pollen percentage, and *Ambrosia:Artemisia*), influenced by the variables listed at the left. Random effects of trap location and year were assessed for significance at *P* < 0.05. Coefficients of the fixed‐effect variables that significantly contribute to each summative metric are listed

	Summative pollen metrics			
	Beta‐diversity	Shannon	NAP%	*Ambrosia:Artemisia*
Fixed effects				
Elevation	–	–	–	–
BareSoil	–	–	–	–
LimestoneRocks	–	–	–	–
BisonManure	–	–	–	–
Bison Density	–	−0.25*	–	–
Area Burned 500 m	–	–	–	–0.14*
Area Burned Flint Hills	0.45***	–	−0.13**	–
Fire Return Interval	–	–	–	–
Mean Fire Return Inter.	–	–	–	–
FireFrequency	–	–	–	–
TimeSinceLastFire	–	–	–	–
Area Burned Konza	−0.29***	–	0.16***	–
Random effects				
Trap	Not significant	Significant (*P*<0.001)	Significant (*P*<0.001)	Significant (*P*<0.001)
Year	Not significant	Significant (*P*<0.05)	Not significant	Not significant

*Indicates the fixed‐effect variable is significant at *P* < 0.05; **Indicates the fixed‐effect variable is significant at *P* < 0.01; ***Indicates the fixed‐effect variable is significant at *P* < 0.001.

**Figure 3:**
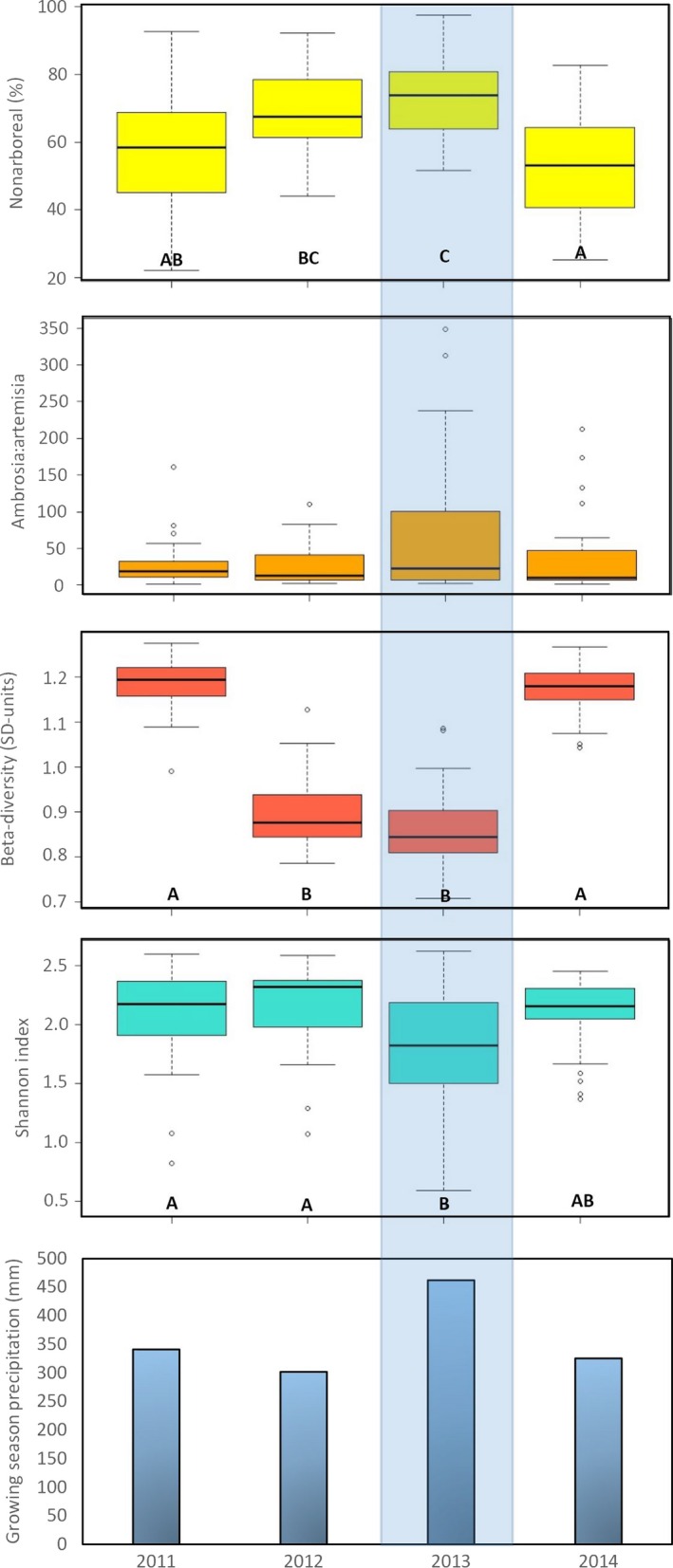
Kruskal–Wallis ANOVA results by year of the four summative metrics, compared to growing season precipitation. Letters (A, B, C, etc.) indicate groupings from the Kruskal–Wallis pairwise comparisons. *Ambrosia:Artemisia* showed no significant difference among the four years of data and have no letters. The blue shading signifies the high precipitation in the 2013 growing season. From top to bottom: Nonarboreal percentage, *Ambrosia:Artemisia*, Beta‐diversity (SD units), Shannon index, and growing season precipitation (mm).

There are significant differences among the 4 years for three of the four summative pollen metrics (Shannon Index, beta‐diversity, and NAP%) demonstrated by the Kruskal–Wallis tests (Fig. 3). However, the amount of variation depends at least to some degree on the metric being used. 2013 stands out as either the highest or lowest year for each of the four metrics. Shannon Index is lowest in 2013 (median 1.82). Beta‐diversity is also lowest in 2013 (median 0.84). Both the NAP% and *Ambrosia:Artemisia* are highest in 2013 (median 73.95 and 23.25, respectively). 2013 is significantly different (at *P *< 0.05) from 2011 in all four metrics. However, it is not significantly different from 2012 in NAP% and beta‐diversity or from 2014 in Shannon Index. 2011 and 2014 were not significantly different from each other in any of the four metrics. In addition, *Ambrosia:Artemisia* did not show any significant differences among the 4 years of data. Total growing season precipitation significantly co‐varies with year for beta‐diversity and NAP% (*P *< 0.05), but not for Shannon Index or *Ambrosia:Artemisia*. Precipitation was highest in 2013 (462 mm) and lowest in 2012 (301 mm).

### Squared‐chord Distance dissimilarity

**Figure 4:**
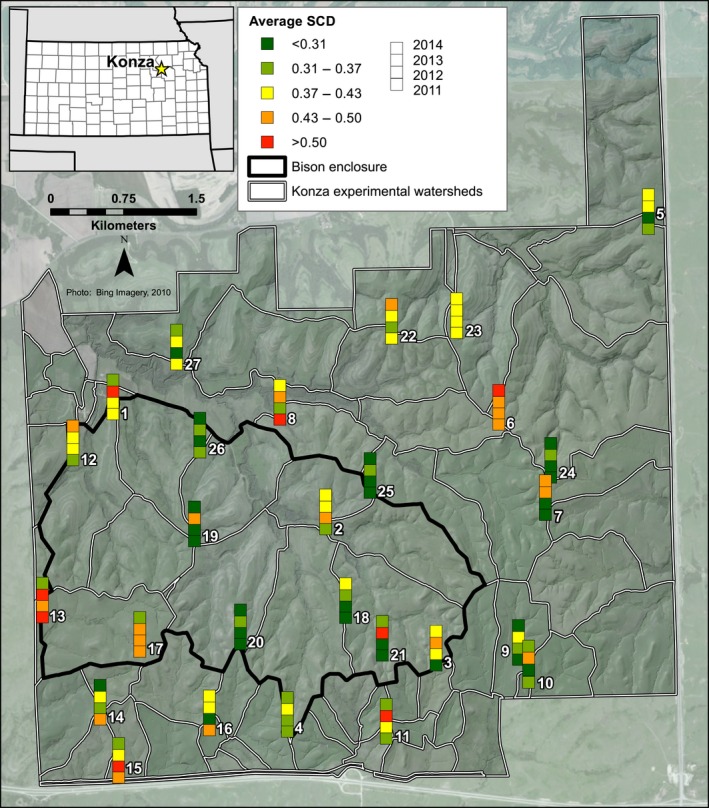
Average squared‐chord distance dissimilarity between a trap sample and all other samples (from all traps and all years). Green values represent lower average dissimilarity, and red values represent higher average dissimilarity.

**Figure 5:**
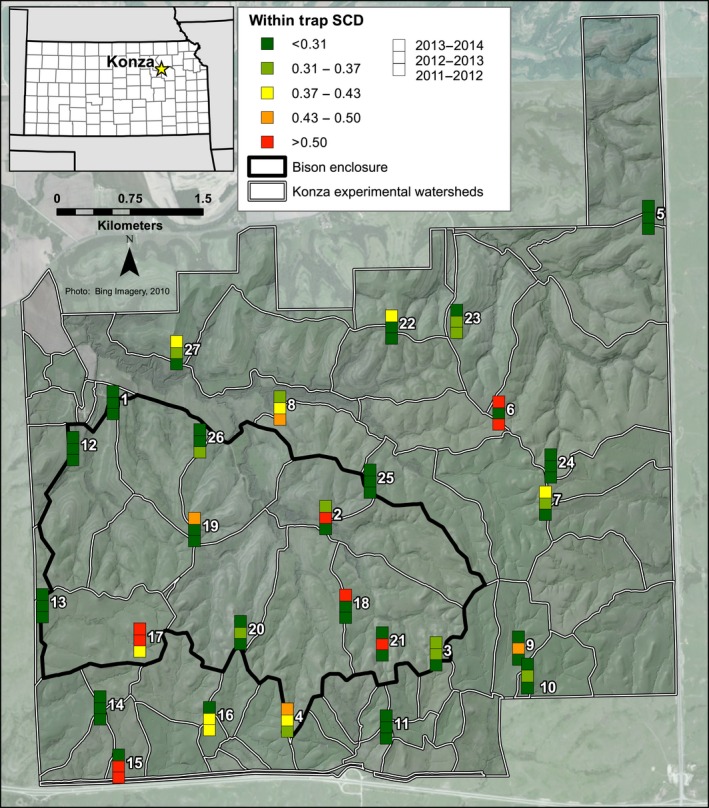
Within‐trap squared‐chord distance dissimilarity from 2011 to 2012, 2012 to 2013, and 2013 to 2014. Green values represent lower average dissimilarity, and red values represent higher average dissimilarity.

**Figure 6:**
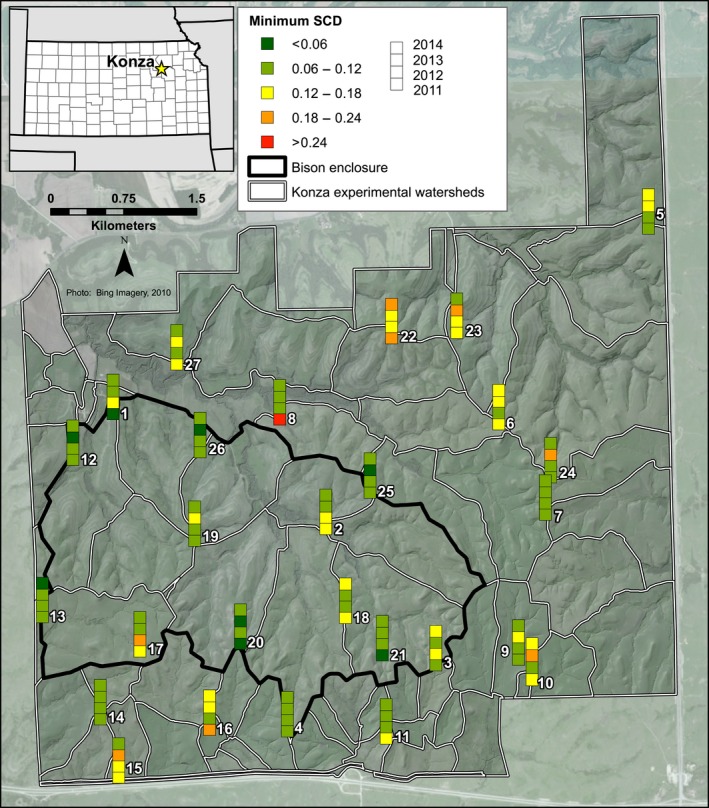
Minimum squared‐chord distance dissimilarity between a trap sample and all other samples (from all traps and all years). Green values represent lower minimum dissimilarity, and red values represent higher minimum dissimilarity. Please note that the color breaks in the legend are different from the breaks used on Figures 4 and 5.

Squared‐chord distance was examined between each sample and all other samples from all traps in all years as an estimation of overall dissimilarity between the multivariate assemblages. The spatial differences in the average dissimilarity between each sample and all other samples across Konza (Fig. 4) are generally higher than the temporal differences for a single trap among the 4 years (Fig. 5). Thus, within‐trap dissimilarity (Fig. 5) is lower than a sample's average dissimilarity to all other samples (across Konza from all 4 years) (Fig. 4). The average dissimilarity between all samples is 0.38, while the within‐trap average dissimilarity is 0.31. Within‐trap dissimilarity is lowest from 2011 to 2012 (average 0.28) and highest from 2012 to 2013 (average 0.33). However, some traps have relatively low within‐trap dissimilarity, while others have high within‐trap dissimilarity (from year to year). For example, Trap 13 has the lowest within‐trap dissimilarity (average 0.11), while Trap 15 has the highest (average 0.70). Minimum dissimilarity for a given sample (the lowest squared‐chord distance value of all pairwise comparisons for a given sample) ranges between 0.01 and 0.30 (Fig. 6), and averages 0.11.

## Discussion

### Influence of fire, grazing, and topography variables

The environmental variables known to influence grassland vegetation – fire frequency, bison density, and elevation – are somewhat reflected in pollen assemblages produced by grassland vegetation. Each of the four summative pollen metrics is influenced by different environmental variables, which is logical given that some of the calculated pollen metrics assess diversity while others focus on pollen composition (Table 1). Previous work in other regions has suggested that there is no single best metric for assessing pollen diversity and composition (Weng et al. 2007; Birks et al. 2016), and our results indicate that the same is true for grasslands in North America. However, some variables (e.g., area burned within Konza and area burned within the Flint Hills) significantly contribute to more than one pollen metric.

The negative influence of bison density on Shannon index is somewhat puzzling (Table 1), as previous work has suggested that bison grazing increases local plant diversity (Hartnett et al. 1996). This could be explained by over‐representation of high pollen producers such as *Ambrosia* and Amaranthaceae in the pollen assemblage, or, under‐representation of low pollen producers such as sunflowers (Asteraceae Undiff.) or Fabaceae family plants (*Amorpha* sp., *Baptisia* sp., or *Lespedeza* sp.) (Commerford et al. 2013). All are common forbs at Konza (Towne 2002). Furthermore, the presence of bison manure (used as a qualitative measure of bison presence/absence) had no influence. This suggests that bison herbivory does have an effect on grassland pollen assemblage diversity, but this signal can only be detected with quantitative herbivore abundance.

Area burned within 500 m of the trap is the only environmental variable that contributed significantly to *Ambrosia:Artemisia* (negative relationship). Higher levels of *Ambrosia* pollen (or lower levels of *Artemisia*, leading to a high ratio) correspond with less area burned. This suggests that grassland fires, at least in a tallgrass prairie like at Konza, may help keep *Ambrosia* abundance or pollen productivity low. This could occur either by fires directly inhibiting *Ambrosia* growth (Abrams and Gibson 1991), or by promoting the growth of grasses and other forbs (including *Artemisia*) in place of *Ambrosia* (Knapp and Seastedt 1986). Additionally, a negative relationship between *Ambrosia* pollen and charcoal was found over the Holocene in the northern Great Plains (Grimm et al. 2011), suggesting that the *Ambrosia*‐fire relationship is robust across different temporal scales.

Although beta‐diversity is influenced by two broader‐scale spatial variables – the area burned within the Flint Hills and the area burned within Konza (Table 1) – this could be partially due to the nature of how the beta‐diversity metric is calculated. Here, we calculated temporal beta‐diversity, in which the value for each sample was the ratio between the sample diversity for 1 year compared to the sample diversity at that same trap for the other 3 years. Because the two above‐mentioned variables are equal across all traps for a given year, it is logical that they would have some influence on the temporal beta‐diversity in this case. However, the lack of influence of the very local, spatial fire variable – area burned within 500 m – is more surprising. The area burned within Konza and the area burned within the Flint Hills operate at a broader spatial scales, indicating that temporal beta‐diversity in our pollen samples is more influenced by annual changes in fires occurring at the local or regional scale than at the very local scale (within 500 m). The influence of regional burning could be manifested directly through fire driving vegetation composition and structure or could suggest that an indirect regional mechanism, such as prevailing wind direction or speed, is influencing pollen dispersal (Davis 2000). Additionally, the other four fire variables – the amount of time since the last fire, fire frequency, mean fire return interval, and fire return interval – had no influence. These variables describe temporal aspects of the fire histories at Konza. This finding is similar to that from Leys et al. (2015), who examined charcoal pieces from these same traps and found that the amount of charcoal was significantly influenced by the area burned within the Flint Hills and Konza, but not by any other fire variables.

Although there is little topographic relief (<100 m) in the study area compared to mountain environments, elevation changes might affect pollen spectra. While elevation did not significantly influence any of the summative metrics at *P *< 0.05, it was very close to contributing to NAP% (*P = *0.057, negative coefficient) and Shannon Index (*P = *0.074, positive coefficient), and so is worth mentioning. In a montane region in Norway, pollen assemblages from a set of traps spanning a larger elevational transect (~500 m) were found to vary greatly in their composition, mostly due to differences in vegetation composition and structure (Birks and Bjune 2010; Bjune 2014). Although the Konza traps span a smaller elevational gradient, the influence of elevation on pollen assemblages here could also be due to systematic differences in vegetation at lower versus higher elevations, although the influence is not significant in the 4‐year record here. Yet, upland soils have been found to support greater vegetation species diversity than lowland soils at Konza (Gibson and Hulbert 1987). Additionally, the lower elevations at Konza contain riparian areas with a greater abundance of arboreal vegetation compared to the upland areas dominated by grass and forbs (Veach et al. 2014). Further, arboreal species tend to be relatively high pollen producers (Brostrom et al. 2008). Thus, the pollen signal of these woody species, particularly *Quercus macrocarpa* and *Quercus muehlenbergii* at Konza, could be amplified in riparian areas where they are already more abundant on the landscape, muting the assemblage diversity assessed by the Shannon Index. It is important to reiterate that the influence of elevation is not statistically significant within the 4‐year record of pollen data presented here. However, additional years of data from these traps could reveal or refute the influence of local elevation differences on these pollen assemblages.

Additionally, the random effects of trap location and year are significant in some of the summative metrics and could be causing the environmental variables (fixed effects) to appear to less significant. The random effect of trap location is significant in Shannon Index, NAP%, and *Ambrosia:Artemisia* (Table 1). This significance indicates that temporal autocorrelation is likely occurring among the 4 years of pollen data within a given trap. This is not completely surprising, as our SCD values within a trap (Fig. 5) were also found to be lower than the SCD values across all traps and years (Fig. 4), indicating higher similarity among the pollen assemblages at a given trap. Vegetation composition is known for being the strongest driver of pollen assemblage composition. While we did not measure vegetation composition as a driver of pollen assemblages, its relationship has been quantified with pollen assemblages in various biomes. At Konza, vegetation varies less at a given trap from year to year and more from trap to trap, so it is logical that pollen assemblages would also vary less at a given trap from year to year. In addition, the random effect of year is also significant in Shannon Index, indicating spatial autocorrelation in Shannon index values (but not in NAP%, beta‐diversity, or *Ambrosia:Artemisia* values) across traps within a given year. This suggests that an annual‐scale driver not included in the regression analyses is influencing Shannon index.

### Annual differences in pollen assemblage diversity

In lacustrine sediment cores, pollen sampling for vegetation reconstruction often does not follow a continuous scheme. Therefore, there is the potential for compositional changes in vegetation (inferred from pollen assemblages) to go undetected during the time between samples (Liu et al. 2012). Beta‐diversity has been calculated on vegetation proxies from lacustrine sediment cores in various regions around the world (Birks 2006; Leys et al. 2014), including pollen from the Great Plains (Commerford et al. 2016), but not on annual modern pollen samples. Our ANOVA results demonstrate statistically significant differences in beta‐diversity between some of the years from 2011 to 2014 (Fig. 3). Yet, despite these differences, our annual averages for beta‐diversity range only between 0.8 and 1.2 SD units. Comparatively, Holocene pollen assemblages from Fox Lake in the northern Great Plains had beta‐diversity values that averaged around 0.8 SD units (4 SD units would imply full turnover), suggesting that grassland vegetation at that site exhibited little change in beta‐diversity over much of the Holocene, despite recurring fire and drought (Commerford et al. 2016). Our beta‐diversity values are similar, suggesting that the amount of change is comparable on an annual time scale.

The pollen data from 2013 are the most extreme of our 4‐year record: lowest beta‐diversity, lowest Shannon diversity, highest *Ambrosia:Artemisia*, and highest NAP% (Fig. 3). This change in the average for all traps in 2013 seems to indicate a regional‐scale driver. Higher precipitation in the 2013 growing season (462.1 mm from May to August, an increase of at least 35% over the other 3 years) is a likely cause. The mechanism could be either through a direct influence on pollen production of certain nonarboreal taxa (McLauchlan et al. 2011) or through increased inwash of pollen from overland flow (Birks and Bjune 2010; Bjune 2014). High summer precipitation has been associated with high levels of *Ambrosia* pollen, while high spring precipitation has been associated with high levels of Poaceae pollen at other monitoring sites in the region (McLauchlan et al. 2011). Given that *Ambrosia* and Poaceae are the two most abundant nonarboreal pollen types in our 4‐year dataset, an increase in their production would certainly affect our summative metrics. *Ambrosia* and Poaceae abundances are highest in 2013 (compared to all other years) at the majority of the traps (Fig. 2), providing further support for the increase being due to production. Higher flowering is a possible instigator of this increase in production (Hicks 2006). However, increased inwash of pollen from overland flow cannot be ruled out as another potential cause. Birks and Bjune (2010) identified inwash as a source of plant macroremains in traps in a Norwegian woodland, and Bjune (2014) also suggested it as a mechanism for pollen deposition. Given the topography of the limestone and mudstone hills at Konza, some of our traps at the lower elevations could be particularly susceptible to pollen deposition via inwash.

In long‐term records of modern pollen, an anomalous year of pollen data (either summarized by a single metric or by individual key taxa) could be muted by the other years of data if averaged over the entire record. Alternatively, a single anomalous year might significantly influence the average if it is substantially different from the other years of data. The role of anomalous individual years could be especially relevant for fossil pollen records from nonlaminated lacustrine sediment cores, where a 1 cm sample often spans a decade or more (depending on the location and the deposition rate; Goring et al. 2012; Minckley et al. 2012a). Ultimately, modern multiyear records that match the time frame of deposition rates in lacustrine cores from the same region could highlight “tipping points” for how anomalous a single year needs to be to affect an average. The longest‐running annual Tauber trap pollen records of which we are aware are in Europe, some of which span 8 years or more (Hättestrand 2013; Pidek et al. 2013; Bjune 2014). In the Great Plains, pollen and sediment deposition rates in lakes are relatively rapid (*e.g.,* 5 years cm^−1^, Grimm et al. 2011) and so fewer years of pollen monitoring would be needed to match their temporal resolution. The medical community has monitored pollen taxa on the Great Plains for decades and has found a direct influence of temperature on pollen production of common allergenic taxa such as *Ambrosia* and *Juniperus* (Levetin 2001; Ziska et al. 2011). Although the collection devices used in those studies are specifically designed to trap airborne pollen rather than deposited pollen – and thus may not serve as a direct comparison to pollen deposited in lake sediment – these records could be sufficiently long to demonstrate the impact of a single anomalous year of pollen production on a multiyear average.

### High dissimilarity among samples

The limited temporal resolution of nonlaminated lacustrine sediment cores has been seen as a potential issue for accurately reconstructing vegetation from pollen assemblages throughout the Holocene (Hicks and Hyvärinen 1999), because it is impossible to achieve an annual‐scale sampling resolution. However, even in the best case scenario, it is likely that sedimentary pollen analysis will not achieve finer than subdecadal resolution (Joosten and de Klerk 2007). Yet, the results of our squared‐chord distance dissimilarity analysis indicate that pollen assemblage composition is more dissimilar across all traps and years (Fig. 4), than it is between years at a single trap (Fig. 5). The relatively lower within‐trap dissimilarity values (year to year) (Fig. 5) indicate that, although significant, annual‐scale differences in pollen productivity or subtle annual changes in grassland vegetation composition may not have as much of an impact on pollen assemblage composition as spatial differences.

Our pollen samples from the Konza prairie have an overall average dissimilarity of 0.38 (the average of the SCD values between all pairs of samples), but an average minimum dissimilarity of 0.11 (the average of the lowest SCD value for all samples). This difference between the overall average and the average minimum dissimilarity between samples indicates that most samples have a close compositional match, but also are quite dissimilar from many of the other samples in our dataset. Our results are similar to those from modern pollen samples from other grassland regions of North America and Africa. In Africa, modern grassland pollen samples were found to have an average minimum dissimilarity of 0.13, while savanna samples were slightly higher at 0.16 (Gajewski et al. 2002). Our Konza prairie samples exhibited slightly lower average minimum dissimilarity (0.11). In Oklahoma and north‐central Texas, tallgrass prairie pollen samples from Tauber traps have also been found to be fairly similar to each other (0.10), but dissimilar to samples from short grass and mixed grass prairie in west Texas and New Mexico (0.40) (Hoyt 2000). Among our Konza prairie samples, the average dissimilarity between all samples is 0.38, which is more comparable to the 0.40 dissimilarity found between the different grassland types by Hoyt (2000). Our samples could be more dissimilar because our traps were intentionally placed to span a range of different burning and grazing treatments to examine the influence of those factors on the diversity and composition of the pollen assemblages. Hoyt (2000) aimed to identify pollen signatures among different grassland types (tallgrass, mixed grass, shortgrass) and therefore did not discuss any variation in burning and grazing, if any, among sample sites.

All of the traps in this study are located within a 3487 hectare area that is predominantly grassland despite some local differences in the amount and type of nearby woody vegetation. Yet, the pollen assemblages from the 27 traps would not all be considered analogs for each other using the Modern Analog Technique with a suggested SCD threshold of 0.20–0.30 (Williams and Shuman 2008). The high variation in these modern pollen assemblages implies that defining a pollen assemblage as “grassland” (particularly when examining fossil pollen assemblages) could be more difficult than previously thought. However, Tauber traps typically detect a very local vegetation signal given their relatively small 5‐cm opening compared to lake surfaces, which have a much larger source area (Calcote 1995; Sugita 2007b). In a lake, the pollen from the entire source area would be mixed together (Sugita 2007a) and so the small source area associated with traps may be a reason for the high dissimilarity among our samples compared to samples from soils or lake sediments. Thus, the mean abundance values from all of the traps would likely be most useful when comparing our samples to grassland fossil pollen records in future work (as suggested by Bjune 2014).

## Conclusion

Modern tallgrass prairie pollen assemblages are more similar temporally than spatially, as shown by SCD values across the 4‐year record. Our SCD values similar to SCD values for pollen samples from modern African grasslands, but higher compared to other modern grasslands in North America. The higher dissimilarity compared to other North American grasslands' pollen samples could be due to our larger sample size, and the deliberate placement of the traps in this study to include a range of elevations, burning frequencies, and grazing intensities. In addition, annual differences in pollen composition and diversity are significant in three of the four summative metrics that we examined: beta‐diversity, Shannon index, and nonarboreal pollen abundance (not significant in *Ambrosia:Artemisia*). Interannual variability in precipitation is significantly related to these differences. The 2013 growing season experienced significantly higher precipitation than 2011, 2012, and 2014, as well as the highest NAP%, lowest Shannon Index, and lowest beta‐diversity of the 4‐year record. Variables of topography, fire, and grazing do have some effect on the diversity and composition of pollen taxa among traps, although their individual significance varies depending on the metric. Further long‐term modern pollen records from grasslands are essential in order to better establish the degree of variation in pollen production and deposition on an annual basis.

## Conflict of Interest

None declared.

## Supporting information


**Appendix S1.** R code for multiple regressions with fixed and random effects, ANOVAs, and squared‐chord distance calculations.
